# Resveratrol Attenuates Learning, Memory, and Social Interaction Impairments in Rats Exposed to Arsenic

**DOI:** 10.1155/2021/9993873

**Published:** 2021-09-28

**Authors:** Zahra Taheri Zadeh, Khadijeh Esmaeilpour, Azadeh Aminzadeh, Mahmoud Reza Heidari, Sara Joushi

**Affiliations:** ^1^Neuroscience Research Center, Institute of Neuropharmacology, Kerman University of Medical Sciences, Kerman, Iran; ^2^Department of Pharmacology and Toxicology, School of Pharmacy, Kerman University of Medical Sciences, Kerman, Iran; ^3^Student Research Committee, Faculty of Pharmacy, Kerman University of Medical Sciences, Kerman, Iran

## Abstract

Arsenic (As) toxicity has deleterious effects on human health causing disorder in the brain. The aim of this study was to investigate the possible neuroprotective effect of resveratrol (RSV) on arsenic-induced neurotoxicity in rats. Neurotoxicity in rats was developed by treating As 10 mg/kg/day for 21 days orally. Animals were put into seven groups: control, vehicle, As, As+RSV10, As+RSV20 mg/kg, RSV10, and RSV20 mg/kg. Behavioral assessments such as the social interaction test, novel object recognition test, elevated plus maze, open field, the Morris water maze, in addition to assessment of biomarkers such as ferric reducing ability of plasma assay, glutathione assay, and malondialdehyde assay, were used to evaluate the effects of RSV on cognitive impairment and molecular changes induced by As. The results showed that cognitive performance impaired in As rats. RSV20 mg/kg significantly could ameliorate behavioral changes like spatial learning in days 3 and 4 (*p* < 0.05), recognition learning and memory (*p* < 0.01), disabilities in motor coordination and stress (*p* < 0.05), increased anxiety (*p* < 0.05), and social interaction deficit (sociability (*p* < 0.001) and social memory (*p* < 0.05)). RSV20 mg/kg also attenuated molecular modifications like decreased antioxidant power (*p* < 0.001), reduced glutathione content (*p* < 0.05), and increased malondialdehyde level (*p* < 0.05) induced by As. In addition to oxidative stress assessments, RSV10 mg/kg could significantly increase FRAP (*p* < 0.01) and GSH (*p* < 0.05); however, MDA was not significantly increased. Our current behavioral findings suggest that RSV has neuroprotective effects against AS toxicity.

## 1. Introduction

Arsenic (As) is known as a global concern that the main route of exposure to this metalloid is through food and water [1]. As influences every organ and tissue in the human body such as the cardiovascular, respiratory, gastrointestinal, productive, and nervous systems [2]. According to previous studies, As affects hippocampal neurons and changes the levels of neurotransmitters and gene regulation, which causes neurobehavioral alterations and neurodegenerative disorders [3]. As claimed by some animal researches, the brain is an important destination of As toxic; hence, it causes impairments on learning and memory that occurred after As accumulation in the brain [4]. Also, As increases 4stress oxidative by generating reactive oxygen species (ROS) which damage proteins, amino acids, DNA, and cell membrane [5]; additionally, a considerable reduction in antioxidant power is evaluated by ferric reducing antioxidant power, and glutathione content [6]. Consequently, malondialdehyde marker is produced by lipid peroxidation and interrupts oxidant/antioxidant balance [7]. According to the study on rats, As impairs social skills and increases anxiety-like behaviors [8]. Also, dopaminergic neurons function is altered in As toxicity since it developed behavioral abnormalities, and some studies have explored its effects on mood disorder such as depression and anxiety [9–12]. Reduced acetylcholine esterase in As-exposed rats expands degeneration of cholinergic neurons that could result in learning and memory impairments [10, 13]. As has been found to disrupt the functioning of N-methyl-D-aspartate (NMDA) receptors and proteins related to postsynaptic and synaptic plasticity in the hippocampus, which regulate learning and memory [14, 15]. In view of boosting the number of As-induced neurotoxicity, the pharmacological ingredient and herbal extracts have been used to assay their protective effectiveness in As-induced neurotoxicity and cognitive impairments [16].

Resveratrol (3,4′,5-trans-trihydroxy-stilbene) is a phenolic compound found in red grapes, raspberries, blackberries, and strawberries [17]. Antioxidant, anticarcinogenic, anti-inflammatory, and antiaging effects of resveratrol (RSV) are well documented [18, 19]. The neuroprotective and cardioprotective effects of RSV are also reported [20]. Furthermore, it is also reported to regulate hepatic metabolism [21] boost, immune system [22, 23], and nervous system [24]. Some studies report the neuroprotective effect of RSV on neurodegenerative disease, which develops due to impaired mitochondrial function [24, 25]. It is found that RSV crosses the blood-brain barrier to fight against oxidative damage in hippocampal neurons and glial cells which results in cognition change [26, 27]. As it is indicated that RSV improves scavenging free radicals and glutathione content, likewise, malondialdehyde level is decreased in consequence to declined lipid peroxidation. Therefore, RSV is able to ameliorate mitochondria redox parameters and improve behavioral alternation [28]. It has been described that RSV may activate silent information regulator 1 (SIRT1), a type of histone deacetylase, to protect neurons against apoptosis, inflammation, and oxidative stress in treatment of neurological disorders such as stroke and Parkinson's disease [29–31]. Also, it regulates cholinesterase activity and protects dopaminergic neurons that improve learning and memory [11, 32, 33]. It is demonstrated that (RSV) is able to reverse social dysfunctions [34]. In addition, some current studies have suggested the effect of RSV in ameliorating panic and anxiety behavior [35]. Our study was designed to investigate the possible protective effects of RSV on cognitive impairments induced by As.

## 2. Material and Method

### 2.1. Animals

Adult male Wistar rats weighing 200 to 230 g were used for the study. The animals were obtained from the animal house facility Kerman University of Medical Sciences. The animals were housed in a room temperature of 22 ± 2°C with a 12 h light : 12 h dark cycle and had free access to food and water.

### 2.2. Experimental Protocol

**Figure 1:**
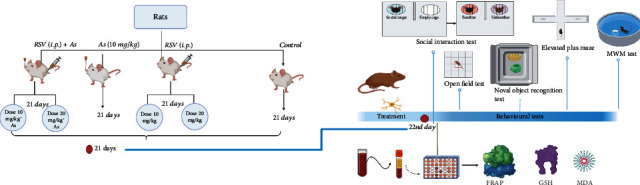
Timeline diagram

Animals were randomly divided into 7 groups (*n* = 8). Group 1 served as a control group and received tap water and food. Group 2 served as a vehicle received normal saline (0.5 ml/kg/day) dimethyl sulfoxide (DMSO) (0.2%) + Ethanol 50%(16 ml/mg) for 21 days. Group 3 received As (10 mg/kg/day) for 21 days [36]. Groups 4 and 5 received As+RSV (10 and 20 mg/kg/day) for 21 days [37]. Groups 6 and 7 received RSV (10 and 20 mg/kg/day) for 21 days only. RSV and vehicle were administered via intraperitoneal (i.p.) injection, and As was administrated orally in drinking water (Figure 1).

### 2.3. Sample Collection

On the 22nd day after the last treatment, phlebotomy was done by a sterile scalpel to collect some drops of blood from a visible vein that was in the underside of the tail, and then blood samples were centrifuged twice in 1500 rpm for 15 minutes and were served in tubes to keep into the refrigerator [38].

### 2.4. Behavioral Tests

On the 22nd day after the last treatment, the behavioral tests like the novel object recognition and open-field tests were performed. From days 23 to 27, the Morris water maze (MWM) was performed. On day 28th, the elevated plus maze and social interaction tests were investigated.

#### 2.4.1. Social Interaction Test

The three-chamber paradigm test is used to study social affiliation and social memory. Social interaction test was done in a box divided into the three compartments with two-wire cages that were placed in the left and right chambers. This test consists of three phases: habituation, sociability, and social novelty test. In the habituation phase, the rat was placed in the central compartment and was allowed to explore the chamber for five minutes.

In sociability phase, the stranger rat 1 was placed in a wire cage, but the another wire cage was empty. The rat was free to explore the three chambers for 10 minutes. In the social novelty phase immediately after the sociability phase, the stranger 2 was added to previously empty wire cage, and it was continued for 10 min. The time spent in each chamber and sniffing time was recorded by a video camera [39].

#### 2.4.2. Novel Object Recognition Test

The novel object recognition test is used to assess recognition memory in rodents. It consists of three phases (habituation, training, and test). In the habituation phase, each rat was allowed to explore the arena freely for 10 min. In the training phase, each rat explored similar objects in the box for 5 min. After 45 min, one of the objects was replaced by a novel object, and the rat was placed into the experimental box again for 3 min. The time spent by rats exploring the objects was measured. The discrimination ratio was calculated in the training and testing phases and is computed as follows: novel/(novel + familiar time). The apparatus and the objects were cleaned between each trial using 70% ethanol to avoid odor trails [40].

#### 2.4.3. Elevated Plus Maze (EPM)

The elevated plus maze test is widely used to assess anxiety-like behavior in rodents. It consisted of two open arms (30 × 5 cm) and two closed arms (30 × 5 × 15 cm) that extended from a central platform (5 × 5 cm). The rats were placed at the center of a maze, and their behavior was recorded during 5 min. Time spent and entries in open arms and close arms were recorded for data analysis.

#### 2.4.4. Open Field

The open-field test is used to evaluate exploratory, locomotor activity, and anxiety-like behavior in rodents.

The open field consists of a plastic box (90 × 90 × 30 cm). The rats were placed in the center of the open-field box, and their behavior was analyzed by using a video-tracking system with a camera for 5 minutes. The duration and frequency of time spent in the center and the number of rearing and grooming were recorded by the Noldus EthoVision system, version 7.1, to measure the locomotive activity of the rats and their level of anxiety-like behavior.

#### 2.4.5. Morris Water Maze

The Morris water maze test was used to assess the spatial learning and memory of the animals. The maze is a circular pool (160 cm in diameter and 80 cm in height) divided into 4 quadrants and filled with water temperature of 25°C. A black square platform (10 cm in diameter) is submerged in northeast quadrant of pool 1.5 cm into water. In the learning phase, rats were trained 4 days to find a platform. Each block consists of four sequential trials. In each trial, the rats were given 60 seconds to find the hidden platform and were allowed to stay on it for 30 s. After the rat found and climbed onto the platform, the trial was stopped. If the rats failed to find the platform within 60 s, they were guided to the platform by the experimenter. Twenty-four hours after the last session, a “probe trial” was applied to assess spatial memory. During this trial, the platform was removed from the maze, and the rat was allowed to search the pool for 60 s. The time spent and distance moved in the target quadrant as well as velocity of movement were recorded by the Noldus EthoVision system version 7.1.

### 2.5. Molecular Assay

#### 2.5.1. Ferric Reducing Ability of Plasma Assay

The ferric reducing ability of plasma assay (FRAP) is used to evaluate antioxidant power. The FRAP assay was performed based on the Benzie and Strain method. It was prepared in 24-well plates, and for each sample, 3 wells were utilized; each well included 0.4 ml of acetate buffer (pH, 3.6) and 50 *μ*l of plasma, and they were kept at 37°C for 2 minutes; then, the reactive mixture that is comprised from 20 mM ferric (III) chloride in 40 mM HCl was added to the wells and rotated for 30 seconds. Finally, absorbance was measured at the wavelength of 593 nm. Additionally, ferrous sulfate pentahydrate FeSO4 was applied for calibration and 5H2O as a standard solution [41, 42].

#### 2.5.2. Glutathione Assay

The spectrophotometric reader assay method is used to evaluate glutathione (GSH) that is operational due to its high accuracy and sensitivity. Firstly, the sulfhydryl reagent 5,5′-dithio-bis (2-nitrobenzoic acid) (DTNB) and 5′-thio-2-nitrobenzoic acid (TNB) were added to samples in 24-well plates that caused GSH oxidation; then, it was incubated for 10 minutes at room temperature, at the end, measured at 412 nm [43].

#### 2.5.3. Malondialdehyde Assay

The thiobarbituric acid (TBA) method is conducted to assess the malondialdehyde (MDA) in biological samples. At first, 1% potassium iodide and 0.1% butylated hydroxytoluene were mixed with samples and passed incubation at 50°C for 20 min. Subsequently, 0.4% TBA was added and incubated at 60°C for 60 min. After the formation of the MDA-TBA complex, isobutyl alcohol was used to extract it, and assessment was done by high-performance liquid chromatography with fluorescence detection [38].

### 2.6. Statistical Analysis

The data was presented as mean ± SEM. For behavioral tests like open field, novel object recognition, and probe test in MWM, comparisons among experimental groups were made by using a one-way analysis of variance (ANOVA). Repeated measures two-way analysis of variance (ANOVA) was used to compare mean differences between groups in the Morris water maze and social interaction test. For statistical significance between groups, Tukey's multiple-comparison posttest was performed. Differences among groups were considered to be significant at a *p* value of < 0.05. Statistical analysis was performed with GraphPad Prism 8.0 (GraphPad Software, Inc., San Diego, CA).

## 3. Result

### 3.1. Effect of As and RSV on Sociability and Social Memory in the Social Interaction Task

**Figure 2:**
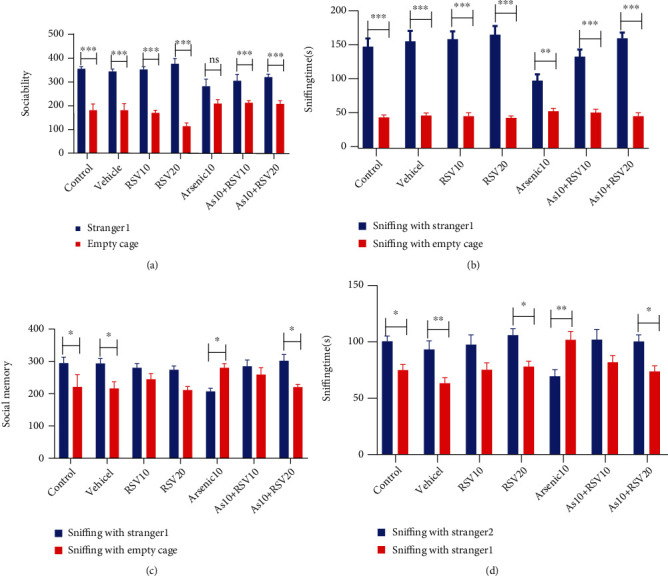
Social interaction task. Sociability: control, vehicle, RSV10, RSV20, As+RSV10, and As+RSV20 groups showed a higher interaction with stranger 1 compared to empty cage. The data are presented as mean ± SEM. ^∗∗∗^*p* < 0.001 vs. control, vehicle, RSV10, and RSV20. ^∗∗^*p* < 0.01 vs. As+RSV10. ^∗∗∗^*p* < 0.001 vs. As+RSV20 (a). There was also a significant difference in sniffing rate with stranger 1 compared to the empty cage in all groups. The data are presented as mean ± SEM. ^∗∗∗^*p* < 0.001 vs. control, vehicle, RSV10, RSV20, As+RSV10, and As+RSV20. ^∗∗^*p* < 0.01 vs. As (b). Social memory: there was a higher interaction with stranger 2 compared to stranger 1 in control, vehicle, and As+RSV20. In As group, rats showed a higher interaction with stranger 1 compared to stranger 2. The data are presented as mean ± SEM. ^∗^*p* < 0.05 vs. control, vehicle, and As+RSV20. ^∗^*p* < 0.05 vs. As (c). There was a higher sniffing rate with stranger 2 compared to stranger 1 in control, vehicle, RSV20, and As+RSV20 groups. There was a higher sniffing rate with stranger 1 compared to the new one in As rats. The data are presented as mean ± SEM. ^∗^*p* < 0.05 vs. control, RSV20, and As+RSV20. ^∗∗^*p* < 0.01 vs. vehicle and As (d).

Social affiliation and social memory were studied by the three-chamber social interaction task: sociability: a two-way ANOVA showed that in As group, there was no statistically significant difference in social interaction between stranger 1 and empty cage. Control, vehicle, RSV10, and RSV20 groups showed a higher interaction with stranger 1 compared to empty cage (*p* < 0.001). There was a higher sociability in As+RSV groups, so that As+RSV10 had a higher interaction toward stranger 1 compared to empty cage (*p* < 0.01) and As+RSV20 also showed significant difference in social interaction between stranger 1 and empty cage (*p* < 0.001) (Figure 2(a)). There was also a significant difference in sniffing rate with stranger 1 compared to empty cage in all groups (*p* < 0.001) except in the As group which shows lower sniffing rate (*p* < 0.01) (Figure 2(b)).

#### 3.1.1. Social Memory (Novelty)

A two-way ANOVA showed that there was a higher interaction with stranger 2 compared to stranger 1 in social memory in control, vehicle (*p* < 0.05), and As+RSV20 groups (*p* < 0.05). In the As group, the rats showed a higher interaction with stranger 2 compared to stranger 1 (*p* < 0.05). There was no significant difference in social memory between stranger 1 and stranger 2 in RSV10, RSV20, and As+RSV(10 mg/kg) groups (Figure 2(c)). There was a higher sniffing rate with stranger 2 compared to stranger 1 in control, RSV20, As+RSV20 (*p* < 0.05), and vehicle rats (*p* < 0.01). There was a higher sniffing rate with stranger 1 compared to the new one in As rats (*p* < 0.05). There was no statistically significant difference in sniffing time between stranger 1 and rat in the As+RSV10 group (Figure 2(d)).

### 3.2. Effect of As and RSV on Recognition Learning and Memory in NOR Test

**Figure 3:**
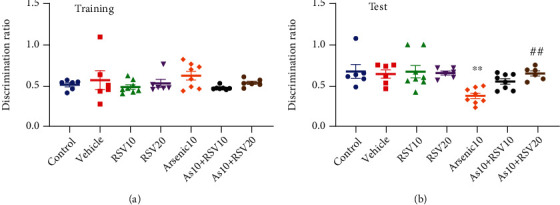
Novel object recognition test. There was no significant difference in discrimination ratio among groups (a). The discrimination ratio in the test phase in As rats is decreased compared to the control group. There was a significant difference in discrimination ratio between As and As+RSV20 groups. The data are presented as mean ± SEM. ^∗∗^*p* < 0.01 vs. control. ^##^*p* < 0.01 vs. As+RSV20 (b).

The NOR test was used to evaluate object recognition learning and memory in experimental groups. One-way ANOVA revealed that there was no significant difference in discrimination ratio among groups (Figure 3(a)). In the test time, the discrimination ratio in As rats was decreased compared to the control group (*p* < 0.05), and a significant difference in discrimination ratio was observed between As and As+RSV20 (*p* < 0.01) in the test phase. There was no significant difference in discrimination ratio between control, vehicle, RSV10, and RSV20 groups in test time (Figure 3(b)).

### 3.3. Effect of As and RSV on Anxiety in EPM

**Figure 4:**
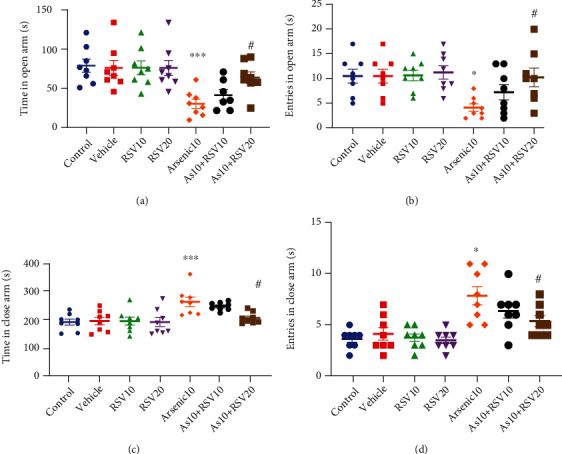
Elevated plus maze. There was a significant difference in time in open arm As and control groups; moreover, there also showed a significant difference in the As group compared to the As+RSV20 group. The data are presented as mean ± SEM. ^∗∗∗^*p* < 0.001 vs. Control. ^#^*p* < 0.05 vs. As+RSV20 (a). There was a significant difference between the As and control groups in entries into the open arm; moreover, there was a significant difference between the As and As+RSV20 groups in this parameter. The data are presented as mean ± SEM. ^∗^*p* < 0.05 vs. control. ^#^*p* < 0.05 vs. As+RSV20 (b). There was a significant difference between the As and control groups in time spent in close arm; moreover, there was a significant difference between the As and As+RSV20 groups in this parameter. The data are presented as mean ± SEM. ^∗∗∗^*p* < 0.001 vs. control. ^#^*p* < 0.05 vs. As+RSV20 (c). There was a significant difference in entries into close arm between As and control groups; moreover, there was a significant difference between As and As+RSV20 groups in this parameter. The data are presented as mean ± SEM. ^∗^*p* < 0.05 vs. control. ^#^*p* < 0.05 vs. As+RSV20 (d).

One-way ANOVA showed that there was a significant difference between As and control group in time spent in open arm (*p* < 0.001) and entries into the open arm (*p* < 0.05). However, As rats significantly spend less time in the open arm and have less entries there, and As groups showed significant differences compared to the As+RSV20 (*p* < 0.05) group in both parameters. But there was no significant difference between As and As+RSV10 groups. There was no significant difference between control, vehicle, RSV10, and RSV20 groups (Figures 4(a) and 4(b)).

A two-way ANOVA revealed there was significant difference between As and control groups in time spent in enclosed arms (*p* < 0.001) and entries into enclosed arms (*p* < 0.001). However, As rats significantly spent more time in enclosed arm and have more entries there, and the As+RSV20 group showed a significant difference compared to the As group in both factors (*p* < 0.05). But there was no significant difference between As and As+RSV10 groups. There was no significant difference between control, vehicle, RSV10, and RSV20 groups (Figures 4(c) and 4(d)).

### 3.4. Effect of As and RSV on Motor Activity and Anxiety-Like Behavior in the Open-Field Test

**Figure 5:**
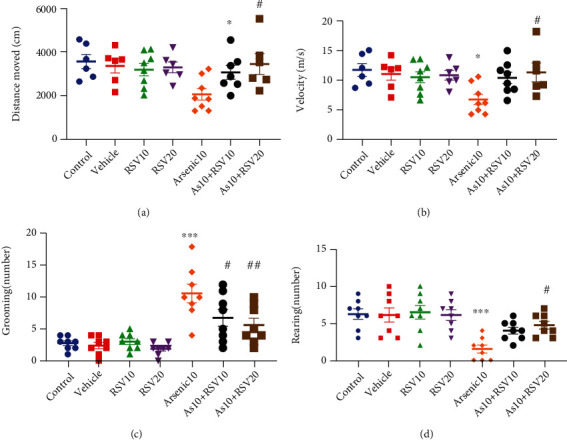
Open-field test. The total distance and the velocity of movement in As rats decreased compared to the control group. There was a significant difference between As and As+RSV20 groups in movement distance and velocity. The data are presented as mean ± SEM. ^∗^*p* < 0.05 vs. control. ^#^*p* < 0.05 vs. As+RSV20 (a, b). In As rats, a significant difference in rearing behavior was observed compared to the control group; moreover, there was significant difference between As and As+RSV20 groups in this parameters. The data are presented as mean ± SEM. ^∗∗∗^*p* < 0.001 vs. control. ^#^*p* < 0.05 vs. As+RSV20 (c). Significant difference in grooming behavior was observed in As rats compared to the controls. There was significant difference between As, As+RSV10, and As+RSV20 groups in this parameter. The data are presented as mean ± SEM. ^∗∗∗^*p* < 0.001 vs. control. ^#^*p* < 0.05 vs. As+RSV10. ^##^*p* < 0.01 vs. As+RSV20 (d).

A one-way ANOVA revealed that both total distances moved, and the velocity of movement was decreased in As rats compared to the control group (*p* < 0.05). There was a significant difference between As and As+RSV20 groups (*p* < 0.05) in movement distance and velocity (Figures 5(a) and 5(b)).

In As rats, a significant difference in rearing behavior was observed compared to the control group (*p* < 0.001); while rearing behavior was decreased in As rats, there was a significant difference between As and As+RSV20 groups (*p* < 0.05) (Figure 5(c)).

A significant difference in grooming behavior was observed compared to the control group (*p* < 0.001); while grooming behavior was increased in As rats, there was a significant difference between As and As+RSV10 (*p* < 0.05) and As+RSV20 (*p* < 0.01) groups in this measurement (Figure 5(d)). There was no significant difference among groups in duration spent in both inner zone and outer zone.

### 3.5. Effect of As and RSV on Learning and Memory in MWM

**Figure 6:**
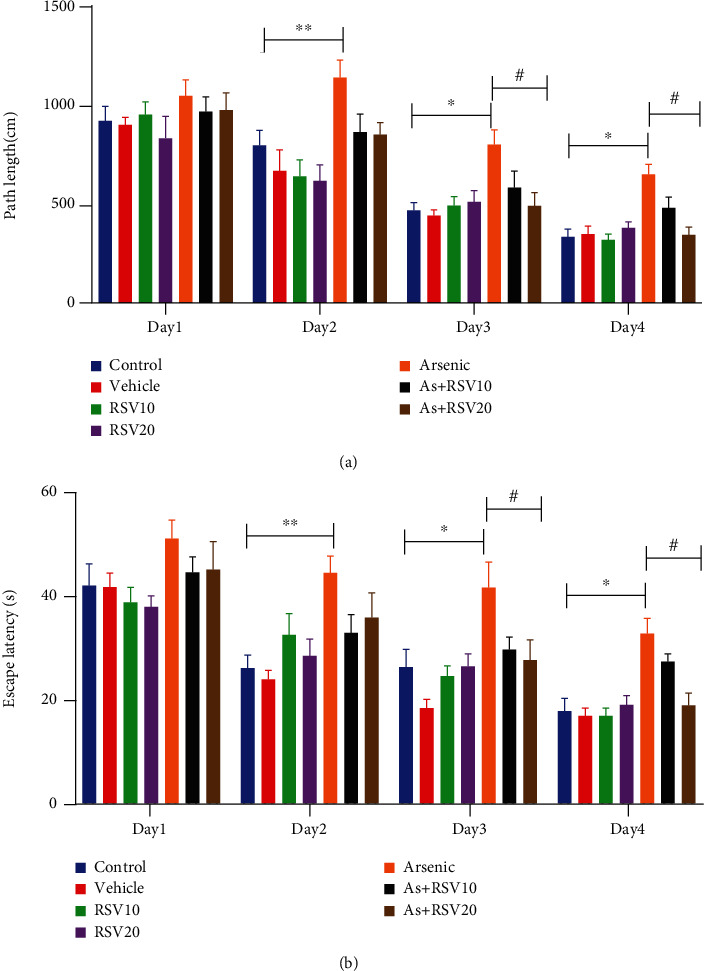
Morris water maze. It was revealed that the total distance moved and escape latency by As rats was increased compared to the control group. There was a decrease in both measurements in the As+RSV20 group compared to the As group. The data are presented as mean ± SEM. ^∗∗^*p* < 0.01 vs. day 2, ^∗^*p* < 0.05 vs. day 3, ^∗^*p* < 0.05 vs. day 4 of the learning phase in the control group. In As+RSV20, ^#^*p* < 0.05 vs. day 3, ^#^*p* < 0.05 vs. day 4 (a, b). There was no significant difference among groups in speed. The data are presented as mean ± SEM (c).

**Figure 7:**
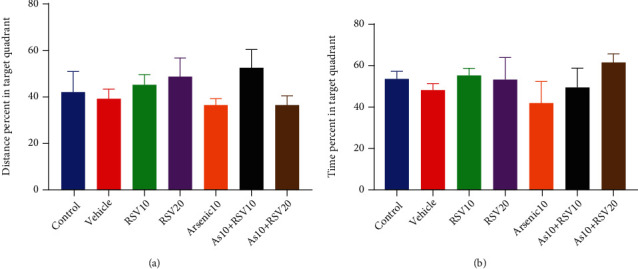
Probe test. There was no significant difference in travel distance and time spent in target quadrant. The data are presented as mean ± SEM (a, b).

The MWM was used to evaluate spatial learning and memory in experimental groups. Learning in the MWM was recorded as a reduction of the swimming path length and escape latency to find the hidden platform during training days. A two-way ANOVA revealed that the total distance moved by As rats was increased compared to the control group in days 2 (*p* < 0.01), 3 (*p* < 0.05), and 4 (*p* < 0.05) of the learning phase. However, there was a decrease in this measurement in the As+RSV20 group compared to the As group in day 3 (*p* < 0.05) and day 4 (*p* < 0.05) (Figure 6(a)). There was a higher escape latency in As rats compared to the control group in days 2 (*p* < 0.01), 3 (*p* < 0.05), and 4 (*p* < 0.05). There was a decrease in this measurement in the As+RSV20 group compared to the As group in day 3 (*p* < 0.05) and day 4 (*p* < 0.05). There was no significant difference between control, vehicle, and RSV (10 and 20 mg/kg) groups (Figure 6(b)). Additionally, there was no significant difference in speed among experimental groups (Table 1). A probe test was conducted 24 h after the last training trial, and the mean percentage of distance and time spent in target quadrant were analyzed to assess spatial memory retention. There was no significant difference among groups in travel distance in target quadrant among the experimental group (Figures 7(a) and 7(b)).

### 3.6. Effect of As and RSV on Antioxidant Power in FRAP Assay

**Figure 8:**
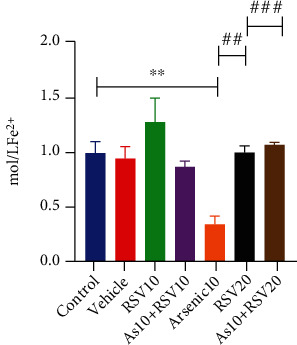
FRAP assay. As and control groups were significantly different in FRAP level; also, As showed a significant difference compared to As+RSV10 and As+RSV20 groups. The data are presented as mean ± SEM. ^∗∗^*p* < 0.01 vs. control. ^##^*p* < 0.01 vs. As+RSV10, ^###^*p* < 0.001 vs. As+RSV20.

The one-way ANOVA showed that there was a significant difference in FRAP level between As and control groups (*p* < 0.01). Moreover, this parameter was significantly different in the As group compared to As+RSV10 (*p* < 0.01) and As+RSV20 (*p* < 0.001) groups (Figure 8).

### 3.7. Effect of As and RSV on Glutathione Content in GSH Assay

**Figure 9:**
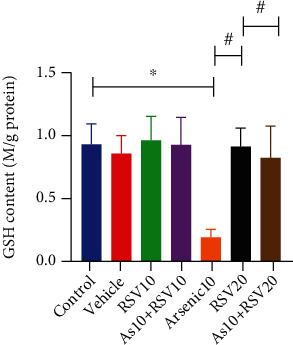
GSH assay. The As and control groups were significantly different in the GSH level; also, As showed a significant difference compared to As+RSV10 and As+RSV20 groups. The data are presented as mean ± SEM. ^∗^*p* < 0.05 vs. control. ^#^*p* < 0.05 vs. As+RSV10, ^#^*p* < 0.05 vs. As+RSV20.

The one-way ANOVA showed that there was significant difference in GSH level between As and control groups (*p* < 0.05). Moreover, this parameter was showed a significantly difference in the As group compared to As+RSV10 (*p* < 0.05) and As+RSV20 (*p* < 0.05) groups (Figure 9).

### 3.8. Effect of As and RSV on Malondialdehyde Level in MDA Assay

**Figure 10:**
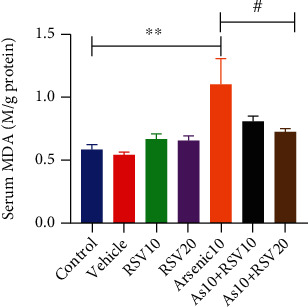
MDA assay. The As and control groups were significantly different in the MDA level; also, As showed a significant difference compared to As+RSV10 and As+RSV20 groups. The data are presented as mean ± SEM. ^∗∗^*p* < 0.01 vs. control, ^#^*p* < 0.05 vs. As+RSV20.

The one-way ANOVA showed that there was a significant difference in MDA level between As and control groups (*p* < 0.01). Moreover, this parameter was significantly different in the As group compared to the As+RSV20 group (*p* < 0.05) (Figure 10).

## 4. Discussion

The purpose of this study was to investigate the effects of RSV (10 and 20 mg/kg/day for 21 days) on cognitive function in male rats after subchronic exposure to As (10 mg/kg). The obtained results of behavioral tests show As exposure causes impairment in motor function, recognition learning and memory, spatial learning, social interaction, and increased anxiety-like behavior. In addition, As diminishes antioxidant capacity and GSH content and increases MDA level due to deterioration of fatty acid in cells membrane. Other studies, which are in agreement with our data, have also shown that acute or chronic exposure to As causes dysfunctions of the nervous system including lower motor speed and impairment in cognition and learning [10, 44]. It is exhibited that cognition and coordination of motor activity are controlled by the cortex, basal ganglia, and cerebellum that As can diminish SOD, GPx, and catalase in these brain parts and induce motor and cognitive dysfunction [45]. Furthermore, animal studies have reported that As is able to accumulate in the mentioned areas of the brain and could induce a variety of neural impairments [46, 47]. It has been demonstrated that chronic exposure of As increases the metabolites of 5-hydroxytryptamine, norepinephrine, and dopamine in the cerebral cortex, hippocampus, and hypothalamus, begins changes in the vertical and horizontal motor activity, and induces anxiety due to MDA, ROS, and cytokines, with increasing result leading to DNA damages [48] [49, 50]. Brain cells achieve their energy throughout the mitochondrial respiratory chain, so the neurons are at risk of stress oxidative, free radicals, and ROS, which disturb DNA, proteins, lipids, and cell membranes [45, 51, 52]. Reduced cell viability and activated caspase-3 result in neuron degeneration which influences brain function [44]. Previous studies also illustrated that As is able to increase lipid peroxidation and decrease significantly the activities of Mn-SOD, CAT, GPX, GST, and GR, which are present in our body to fight ROS, but overproduction of ROS in As poisoning makes an antioxidant imbalance which causes a decrease in the mitochondrial membrane potential, which induce production of proinflammatory cytokines and apoptosis factors [44, 53]. GSH as the most important antioxidant factor plays a critical role to protect neurons from stress disturbances; meanwhile, As and interactions with GSH contents and related enzymes have connected changes with neurodegenerative disorders such as Alzheimer's disease, schizophrenia, and Parkinson's disease [54], because of reduced GPx and glutathione reductase that convert GSH to GSSG form. On the other hand, another investigation has exhibited poor social behavior in mice which had long-term consumption of a special diet containing As, and more research have proven that As exposure is able to modify social behavior by regulating serotonin receptors, reducing BDNF (brain-derived neurotrophic factor) [55, 56], and increasing inflammatory and oxidative compounds that cause changes in mood owing to alternations in gene expression and synaptic plasticity [56]. Adenosine monophosphate-activated kinase (AMPK), which control cellular energy balance and neuronal development, is affected in As toxicity and may cause neurodegenerative diseases [57]. Moreover, As induces autophagy by inhibiting PI3K (abstract phosphatidylinositol-3kinase) signaling pathway that leads to spatial learning dysfunction; meanwhile, overproduction of oxidative factors and ROS could decrease GSH, FRAP, and GPx and develop impairment of learning and memory [56] [9]. As has been illustrated to induce inflammation by activating ERK1/2 (extracellular signal-regulated kinase1/2), JNK (c-Jun amino-terminal kinase), and mitogen-activated protein kinase (MAPK) pathways that could induce neurodegenerative diseases and behavioral alternation [9, 58, 59]. Hippocampal neuronal apoptosis through an upregulated BMP2 (bone morphogenetic proteins) attenuation induces cognitive deficits in As poisoning [60].

RSV, a common phytoalexin compound, is known as an antioxidant, anti-inflammatory, and neuroprotective component. The behavioral tests in our studies showed that RSV (20 mg/kg) has improved motor dysfunction, impaired recognition learning and memory, and decreased spatial learning, poor social interaction, and anxiety-like behavior. RSV is able to scavenge free radicals (including reactive oxygen species/reactive nitrogen species (ROS/RNS)) that damage vital molecules in cells due to oxidative stress and enhances cellular antioxidant defense [61, 62]. RSV increases antioxidant defense through the induction of antioxidant enzymes synthesis including GPX, SOD, glutathione S-transferase, and declining lipid peroxidation by increasing GSH content that include sulfhydryl group which performs as a scavenger [46, 63, 64]. .Some research has proved that RSV exerts anti-inflammatory effects by the suppression of IL-1 and IL-6, reduction of the expression and activity of COX-2, and inhibition of TNF, histamine, and PG synthesis which play an important role to commence inflammation [65, 66]. According to recent findings, RSV is able to reverse neurobehavioral and recognition memory impairments and motor deficits through promoting the sirtuin1 (SIRT1) and AMPK activation [67–70]. Moreover, it is demonstrated that RSV decreases the generation of free radicals and elevates oxidant defense system by generating GSH content that attenuates motor impairment and pathological alternations [30]. Other studies in rats have shown that resveratrol pretreatment significantly improves spatial learning and memory impairments that were made by A*β*1-42 [71] and decreases hippocampal content of MDA. It also increases SOD activity in rats suffering from vascular dementia and GSH level in senescence-accelerated mice and consequently improves their spatial learning and memory ability [72] [73]. Previous studies have demonstrated that RSV decreases anxiety-like behavior by improving increased glycogen synthase kinase-3 (GSK-3*β*) levels in rats with metabolic disease [74]. Additionally, RSV declines stress and depression mood in rats by improving GSH, SOD, and catalase content and decreasing MDA, lipid peroxidation, and corticosterone [75]. More investigations have demonstrated that social deficits were prevented in the animal models of autism by RSV [76]. Furthermore, other research has exhibited that coadministration of resveratrol and astaxanthin improves critical and effective factors in defense antioxidant system including SOD, CAT, and GSH; however, MDA and NO molecules are suppressed in some kind of models of autistic mice to enhance sociability [77]. In addition, resveratrol is able to protect the hippocampal neurons, decrease inflammation and oxidative stress, and improve cognitive impairments by regulating the Janus kinases, extracellular signal-regulated kinases, and signal transducers and activators of the transcription (JAK/ERK/STAT) signaling pathway in rats with cerebral ischemia-reperfusion injury [78]. Moreover, RSV has delineated the improvement in recognition and spatial learning in the model of mice with depression [79].

## 5. Conclusion

RSV20 mg/kg improved cognitive functions such as memory and learning, sociability, and anxiety-like behavior in As-induced neurotoxicity. Further studies are warranted aiming at molecular mechanism.

## Figures and Tables

**Table 1:** Velocity in the MWM task.

	Control mean ± SEM	Vehicle mean ± SEM	RSV10 mean ± SEM	RSV20 mean ± SEM	Arsenic 10 mean ± SEM	RSV10+AS mean ± SEM	RSV20+AS mean ± SEM
Velocity (cm/s)							
1st day	25.26 ± 3.91	22.26 ± 1.01	24.08 ± 4.99	22.91 ± 3.00	20.99 ± 0.94	22.90 ± 1.43	21.84 ± 1.14
2nd day	28.74 ± 4.00	23.89 ± 4.81	22.81 ± 2.79	22.20 ± 2.16	27.89 ± 4.22	23.52 ± 1.42	26.27 ± 3.79
3rd day	21.82 ± 2.91	24.69 ± 1.36	22.81 ± 1.47	21.86 ± 4.43	25.43 ± 7.10	29.21 ± 4.33	23.82 ± 4.39
4th day	22.35 ± 3.86	23.77 ± 3.80	21.14 ± 1.36	22.28 ± 2.65	20.55 ± 1.80	28.31 ± 2.21	20.17 ± 1.65

## Data Availability

The data that support the finding of this study are available from the corresponding author upon reasonable request.
